# Open-Source Large Language Models and AI Health Equity: A Health Service Triangle Model Perspective

**DOI:** 10.2196/86769

**Published:** 2026-04-29

**Authors:** Shaolong Wu, Wenxin Zou, Jiong Tu, Chunxiao Wang, Cheng Jin, Jing Liao, Kwok Cho Tang, Ning Liu, Chun Hao

**Affiliations:** 1 Department of Public Administration School of Government Sun Yat-sen University Guangzhou China; 2 Sun Yat-Sen Chinese Public Management Research Center Sun Yat-sen University Guangzhou China; 3 Sun Yat-sen Global Health Institute Institute of State Governance Sun Yat-sen University Guangzhou, Guangdong China; 4 School of Sociology and Anthropology Sun Yat-sen University Guangzhou China; 5 School of Health Management Guangzhou Medical University Guangzhou China; 6 Party Committee Huizhou Municipal Central People's Hospital Huizhou China; 7 Department of Medical Statistics School of Public Health Sun Yat-Sen University Guangzhou China; 8 Department of Public and International Affairs City University of Hong Kong Hong Kong China (Hong Kong); 9 School of Management Lanzhou University Lanzhou China; 10 China Research Center for Government Performance-Management Lanzhou University Lanzhou China

**Keywords:** artificial intelligence health, AI health, open-source large language models, open-source LLMs, health equity, health service triangle model, democratization, artificial intelligence, AI

## Abstract

This study explores the role of open-source large language models (LLMs) in promoting artificial intelligence (AI) health equity from the perspective of the health service triangle model. First, it defines AI health, categorizes AI-supported decision-making patterns, and assesses the status quo of AI health inequalities. Second, by comparing open-source and closed-source LLMs in terms of patient privacy, data security, accessibility, and use, it demonstrates the distinct advantages of open-source LLMs for AI-enabled health services. Finally, based on the health service triangle model, this study demonstrates how open-source LLMs drive the democratization of AI-enabled health services—particularly benefiting low-resource regions—by expanding service types, improving accessibility, enhancing quality, and reducing costs. This study concludes that, while open-source LLMs must address challenges such as hallucination risks and ethical responsibilities, they ultimately enable AI health equity through technological sharing.

## Introduction

Artificial intelligence (AI) typically refers to computational technologies that mimic the mechanisms of human intelligence [1-4], such as thinking, deep learning, adaptation, interaction, and understanding [5]. Alternatively, it can be defined as a computational program or machine performing a task that, if accomplished by a human, would be considered intelligent [6]. AI technologies are being extensively used in the health sector to tackle the myriads of challenges confronting it. We use the term “AI health” to describe the application of AI technologies in the health sector to influence health outcomes. The fundamental distinction between AI health and digital health lies in the fact that, in the former, decisions and actions directly related to health (at both the individual and population level) are made or assisted by AI (computer programs and machines) rather than being independently made by humans. In other words, the defining characteristic of AI health is that AI now assists—or, in some cases, even replaces—human decision-making in matters of health. This represents an unprecedented development in human history.

**Figure 1:**
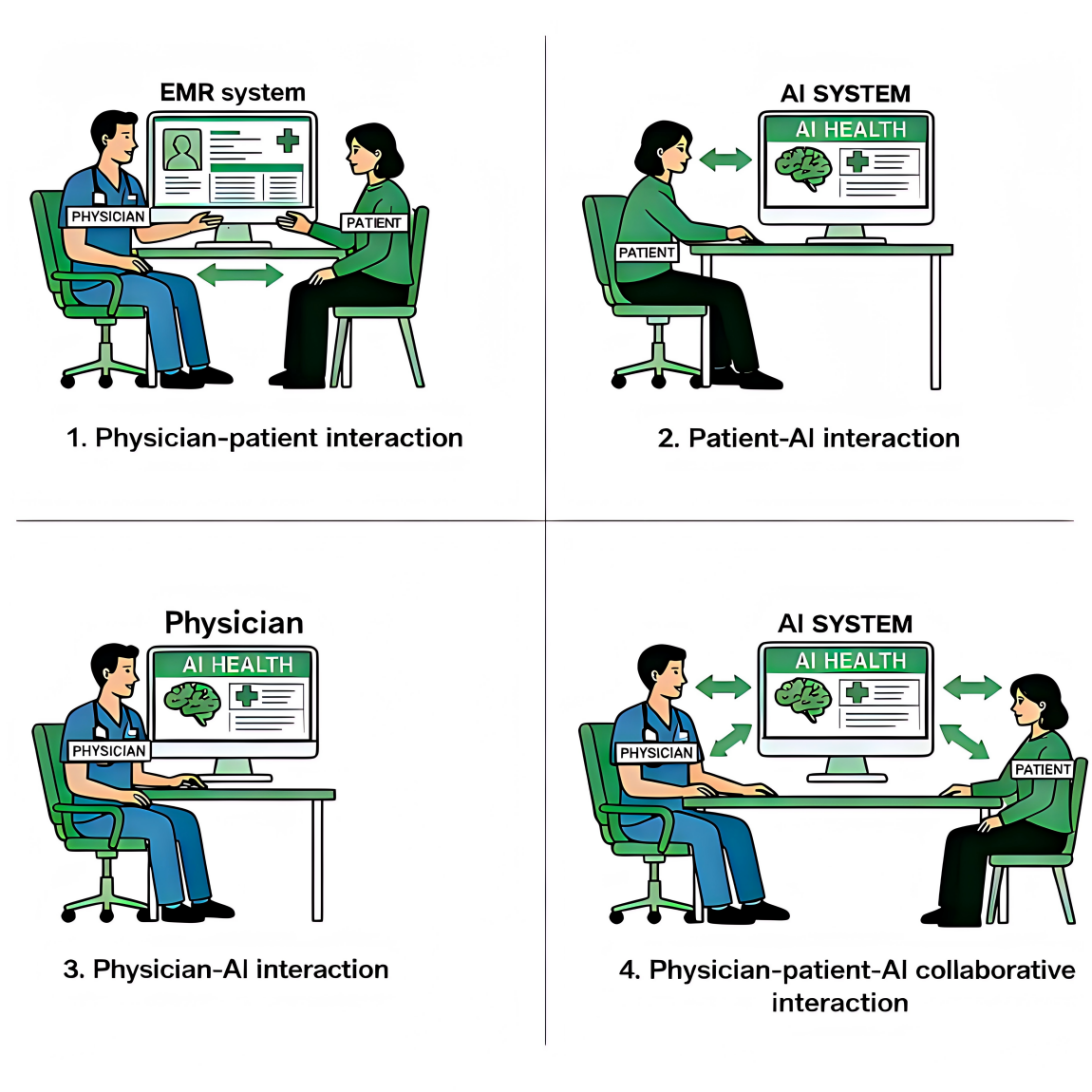
Four types of physician–patient–artificial intelligence (AI) decision-making patterns. EMR: electronic medical record.

In the era of AI-assisted health care, decision-making patterns can be categorized into 4 types based on the extent of AI involvement in the health care process (Figure 1): traditional physician-patient interaction (in which physicians make diagnostic and treatment decisions without AI support relying solely on patient-provided information and conventional examinations), patient-AI interaction (where patients use AI tools to support their own health care self-management decisions), physician-AI interaction (in which physicians use AI for clinical decision support), and physician-AI-patient collaborative interaction (where physicians and patients jointly use AI to facilitate shared decision-making throughout the diagnostic and treatment process) [7,8].

The transition from digital health to AI health represents a paradigm shift in health care, evolving from a physician-patient dyadic model to a triadic physician-patient-AI interaction model. This transformation constitutes an inevitable trend as it holds significant potential to address systemic challenges such as reducing unaffordable costs for patients, alleviating excessive workloads and physician burnout, and enhancing health care quality and operational efficiency [9]. By advancing toward a more integrated, intelligent, and human-centered system, this shift can ultimately contribute to better health outcomes and sustainable well-being [10].

However, the development of AI technology is uneven across countries, which has led to disparities in its application in the health care sector, resulting in inequalities in health services and health outcomes [11]. According to the Artificial Intelligence Index Report 2025, the United States leads in AI model development with 40 models in 2024 and a dominant cumulative output over the past decade. China produced 15 models in 2024, with its leading model (eg, DeepSeek) achieving near–state-of-the-art performance. Europe released only 3 models, continuing a declining trend, whereas regions such as the Middle East, Latin America, and Southeast Asia contributed sporadically and minimally [5]. In terms of research on AI applications in health care, the United States holds significant influence, and AI is widely used in specialized fields such as radiology and pathology, optimizing clinical decision support systems. Following the release of the DeepSeek model, many of China’s hospitals have deployed its public version [12]. In other countries, AI model development remains at an early stage and relies more heavily on international collaboration, resulting in only sporadic outputs; a similar pattern is seen in AI health applications, to which these countries have so far made only limited contributions [5].

In addition to gaps in AI research and health care deployment, access to and use of these technologies remain unequal across demographic groups. As the most advanced frontier of AI, large language models (LLMs) are predominantly controlled by a few leading companies and are close sourced to the public or health care providers. Given the restricted access to these LLMs, the resulting AI health applications are also typically released only to specific user groups by their developers. Similar to gaps observed in digital health [13-15], AI health inequalities stem from disparities in computational power (eg, graphics processing unit [GPU] chips), data resources (eg, standardized datasets), and algorithms (eg, LLM training frameworks). Gaps in access to AI health applications, differences in application skills, and uneven opportunities for use further exacerbate AI health inequity [16,17]. However, with DeepSeek leading a new wave of open-source initiatives [12], the widespread adoption of LLMs has the potential to fundamentally improve the foundation of AI health equity.

Why and how do open-source LLMs, compared to closed-source LLMs, improve AI health equity? Investigating this question helps policymakers, LLM companies and developers, hospitals, physicians, patients, and public health professionals understand the role and trends of AI in the health domain and actively promote the equitable access and application of open-source LLMs in health care. On the basis of a conceptualization of AI health and an analysis of the factors influencing AI health inequality, this study conducted a comparative analysis of the advantages of open-source LLMs over closed-source LLMs in improving AI health equity.. Specifically, using the health service triangle model as an analytical framework, this study compared open-source and closed-source LLMs across health service types, accessibility, quality, and cost. It argues that the local deployment of open-source LLMs offers unparalleled advantages in protecting patient privacy and data security, which in turn facilitates the development of more diverse health services, improves accessibility, enhances quality, and reduces costs. While open-source LLMs face challenges such as hallucinations, technology acceptance, and ethical issues, they are ultimately expected to improve AI health equity through technology sharing.

## Closed-Source and Open-Source LLMs and AI Health Equity

The development of AI has historically followed 2 distinct paths: closed-source and open-source. The fundamental tension between these approaches reflects a divergence between technological democratization and commercial monopolization. The closed-source camp advocates for maintaining proprietary control over technology to ensure service quality, achieve commercial returns, safeguard model security and intellectual property, and optimize model performance. In contrast, the open-source approach promotes the sharing of model algorithms, code, and data to engage global developers in improving the models, thereby accelerating innovation and broadening accessibility.

In the early stages of LLM development, many academic institutions and small to medium enterprises embraced open-source principles. However, as LLMs advanced, major technology giants and unicorns increasingly adopted closed-source strategies. Although some leading firms—such as Meta with its Llama series—have pursued open-source releases, their models often lag in performance compared to proprietary alternatives and have not significantly challenged the dominance of closed-source approaches. However, with the emergence of high-performance open-source models such as DeepSeek, the global AI industry is experiencing an unprecedented wave of open-source innovation. Table 1 provides detailed comparisons.

**Table 1:** Open-source status of major large language models (compiled by the authors from publicly available information [retrieved via Google] as of August 31, 2025).

Model name	Publisher	Parameter scale	Open source announcement date
Gemma	Google DeepMind	2B-7B^a^	February 21, 2024
Mistral 7B version 0.2	Mistral AI	7B	March 24, 2024
DBRX	Databricks	132B	March 27, 2024
Nemotron-4	NVIDIA	340B	June 14, 2024
Llama 3.1	Meta	8B-405B	July 23, 2024
DeepSeek-V3	DeepSeek	671B	December 26, 2024
MiniMax-01	MiniMax	456B	January 15, 2025
DeepSeek-R1	DeepSeek	1.5B-660B	January 20, 2025
Qwen2.5-1 M	Alibaba Cloud	7B-14B	January 27, 2025
Gemma 3	Google DeepMind	1B-27B	March 12, 2025
Llama 4	Meta	109B-2T^b^	April 5, 2025
Qwen3	Alibaba Cloud	0.6B-235B	April 29, 2025
GPT-OSS-120B	OpenAI	120B	August 6, 2025
GPT-OSS-20B	OpenAI	20B	August 6, 2025

^a^Denotes billion parameters.

^b^Denotes trillion parameters.

The openness of LLMs can be categorized into 3 levels: fully open-source, partially open-source, and planned open-source. Fully open-source models not only release model weights but may also include training code, datasets, inference deployment solutions, and more. They allow individuals, enterprises, and institutions to use the models freely, including for commercial purposes, without requiring additional authorization. DeepSeek’s series of models are fully open-source, permitting free use, local deployment, closed-source fine-tuning, and commercial applications. Fully open-source models have emerged as a key enabler for democratizing LLMs and fostering innovation in health applications. By lowering the barriers to accessing advanced LLMs, open-source initiatives allow diverse stakeholders—from regional hospitals to community clinics and individuals—to develop and deploy tailored health solutions. This accessibility helps reduce disparities in health care capabilities across different countries and demographic groups, thereby promoting health equity through broader and more equitable adoption of AI health technologies.

The direct factors influencing health include demographic and biological factors, environmental factors, behavioral factors, psychological factors, and health care factors [18,19]. The application of AI can impact most of these factors, thereby exerting a broad influence on health—a concept we define as “AI health,” denoting the application of AI technologies within the health sector to shape health outcomes. AI health equity involves multiple levels and dimensions, ranging from individual to regional scales and from behavioral to health care factors. Inequities in foundational resources (computational power, data, and algorithms) and throughout the AI life cycle (research and development, application, access, and use) within health care drive disparities in health outcomes. This constitutes AI health inequity. Thus, compared to closed-source LLMs, open-source LLMs demonstrate greater potential to reduce these disparities and contribute to enhanced AI health equity. At present, AI primarily affects health care services, improving health through health education and promotion, prevention, treatment, and rehabilitation. Given that the impact of AI on health is a relatively recent development and the significant influence of open-source LLMs on health only emerged in early 2025, this paper focuses specifically on the impact of LLMs on health service equity.

For the health service industry, ensuring data security and patient privacy remains one of the most important concerns when applying LLMs [20-22]. A significant advantage of fully open-source LLMs is that they can be deployed locally, enabling health care institutions to maximize the protection of patient privacy and data security [21,23-26]. Therefore, for both the demand side (eg, patients and health care providers) and supply side (eg, developers and institutions), open-source LLMs can help address health service inequalities often associated with closed-source LLMs. By overcoming these key barriers, open-source LLMs pave the way for enhancing the scope, quality, and affordability of health services, contributing to a more comprehensive improvement in AI health equity on a global scale.

## Framework for AI Health Equity: Health Service Triangle Model

**Figure 2:**
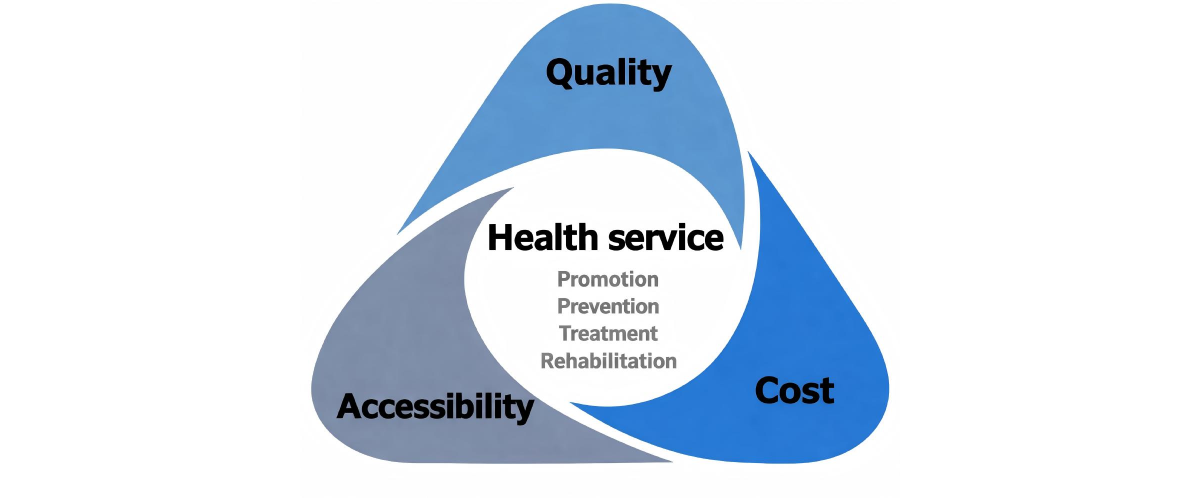
Health service triangle model.

In the field of health services, 2 models are widely recognized: one is the World Health Organization’s health system model focusing on the health care supply side [27], and the other is the model by Andersen [28] emphasizing the health care demand side. By integrating both supply and demand perspectives, Wang et al [29] developed a health service triangle model based on the essential common elements of these 2 frameworks to analyze online health services (for details, refer to Figure 2). Furthermore, this model can also be applied to evaluate the impact of open-source LLMs on AI health equity.

The health service triangle model highlights health services and their 3 indispensable fundamental elements: accessibility, quality, and cost. Health services form the core of the model, and the type of service determines its accessibility, quality, and cost. What kinds of health services can LLMs actually help provide? Without health services, there is no AI health—the type of health service is the foundation for evaluating AI health equity. For instance, emergency and urgent care services prioritize accessibility. If quality and cost are prioritized instead, delayed treatment could lead to severe health consequences. Accessibility includes geographic, temporal, economic, social, and eligibility dimensions [30,31]. Certain health services delivered via LLMs through online channels hold significant advantages in accessibility. Users can overcome spatiotemporal constraints, accessing services anonymously, inexpensively, or even free of charge around the clock [29].

Health service quality refers to the extent to which health care professionals and health care organizations deliver care to individuals and populations in ways that align with the best expected health outcomes achievable given current medical knowledge and technology [32]. LLMs have the potential to significantly enhance diagnosis, prescription, and treatment and are expected to address issues such as physician shortages, substandard care, and medical errors. The rapid rise in health care costs, which often makes care unaffordable for patients, has long been a fundamental barrier to the sustainable development of health systems. LLM technology, capable of efficiently processing medical images, assisting in diagnosis, discovering drugs, and improving medical education and research, is also seen as a promising tool to curb escalating medical expenses [9]. In terms of indirect costs—such as travel expenses—LLM-powered telemedicine can significantly reduce these burdens.

On the basis of the nature of health services and their triangular attributes, we established a comparative analysis framework for closed-source and open-source LLMs (for details, refer to Table 2). Compared to closed-source LLMs, open-source LLMs generate more positive social outcomes in terms of health service types, accessibility, quality, and cost—leading to a fairer distribution across regions and populations. In summary, the advantages of open-source LLMs over closed-source ones—in terms of health services and their 3 core attributes—enable AI to be widely used to meet various health service needs of both providers and users. This is particularly beneficial for remote and rural areas, financially constrained institutions, and privacy-sensitive applications. While closed-source LLMs can only offer limited generalized services targeted at users with payment capacity, open-source LLMs advance AI health equity by broadening access through more diverse and affordable services.

**Table 2:** Comparative analysis of artificial intelligence health equity: closed- and open-source large language models (LLMs).

Health service	Closed-source LLMs	Open-source LLMs
Types	Limited availability	Wider variety
Accessibility	Restricted (API^a^ dependent and paywall)	Flexible (local deployment and customizable)
Quality	Consistent but generic	Variable but adaptable
Cost	Recurring fees (pay per use)	Low licensing cost and high setup cost

^a^API: application programming interface.

This study used a multimethod approach to compare open-source and closed-source LLMs across 4 dimensions of health services: types, accessibility, quality, and cost. Data were collected through web-based surveys; document research; and a systematic literature review, which included 31 AI model web pages (2020-2025), 7 DeepSeek technical documents, and 80 academic publications. The analysis quantified the relative strengths and weaknesses of each model type, demonstrating how the open-source paradigm promotes the use of AI-supported health services and contributes to improved health outcomes. The analysis shows that open-source LLMs are rapidly empowering both supply-side (eg, developers and institutions) and demand-side (eg, patients and health care professionals) actors, especially those in low- and middle-income countries (LMICs), to adopt AI in addressing their respective challenges. As AI-enabled health services become more widespread, this trend supports the achievement of AI health equity on a broader scale.

## Comparative Analysis of AI Health Equity: Closed- and Open-Source LLMs

### Service Types

With its advancement into the generative AI era, AI appears capable of performing nearly all tasks that humans can accomplish. In the health care sector, almost all health-related decisions and tasks seem achievable or assistable through LLMs. China’s National Health Commission released the Reference Guide for Artificial Intelligence Application Scenarios in the Health Industry in 2024, summarizing AI applications across 13 categories and 84 specific scenarios in the health sector. The 13 application categories include medical services, pharmaceutical services, health insurance services, traditional Chinese medicine management, hospital administration, health management, public health services, older adult care and childcare, medical robots, drug development, traditional Chinese medicine industry, medical education, and medical research. The 84 scenarios further break down each category into specific application areas [33].

In other countries, AI applications in the health sector—primarily in medical diagnosis and decision support (eg, disease classification and prediction, clinical reasoning and planning, and dynamic referral), medical image processing, electronic health record and text analysis, bioinformatics and drug development, public health, and medical education [2,34-36]—vary according to local health challenges and system structures. Despite the broad range of applications, medical diagnosis and treatment remains the core function of AI in health care. However, it is important to note that most AI applications in health care are still in the research and testing phases and have not yet been widely integrated into routine clinical practice [37]. With the rise of open-source LLMs and their local deployment, many of these experimental applications are gradually being implemented in real clinical settings [38]. AI-supported health services, such as other health technologies throughout history, are expected to eventually achieve widespread adoption and popularization.

For individual users, there is no fundamental difference in the types of health services provided by closed-source and open-source LLMs. However, for enterprises and health care institutions, closed-source LLMs (such as GPT-4) are constrained by vendor limitations. Even when used for developing professional health care software, the range of services they can offer remains restricted. In contrast, open-source LLMs (eg, DeepSeek-R1) can be used commercially without vendor-imposed limitations, enabling health care organizations to deploy them locally and develop a wide variety of specialized or personalized health services tailored to local contexts. This flexibility overcomes the application programming interface (API) dependencies and paywalls inherent to closed-source LLMs, allowing institutions to better address specific health care needs.

### Accessibility

Accessing closed-source LLMs via the internet typically requires account registration and payment. Some leading LLM companies impose IP blocks on specific countries, preventing users from those regions from registering or using their services. Credit card payments may be prohibited, and API calls might be restricted or return simplified results. Certain countries have also implemented policies to restrict the export of key technologies related to LLMs—such as chips and model weights—to maintain their technological advantage and monopolistic position [39]. In contrast, fully open-source LLMs, such as those released by DeepSeek—which provide both model weights and code, along with distilled versions ranging from 1.5B to 671B parameters—enable worldwide access. Institutions and individuals worldwide can not only directly use these models but also deploy them locally if they possess sufficient computational resources. This increases the likelihood that patients will be able to access AI-provided or AI-assisted decision-making to address their medical needs. In other words, both health care supply-side and demand-side stakeholders benefit from the improved accessibility enabled by open-source LLMs.

Open-source LLMs are typically released under open licenses such as the MIT License or Apache License 2.0, permitting unrestricted commercial and research use. After local deployment, the transparency of model weights and architecture enables autonomous modifications, allowing for secondary development tailored to specific domains—such as model distillation and fine-tuning to adapt to particular health care scenarios and tasks [40]. In addition to enabling offline use, open-source LLMs crucially avoid the need to upload data to external models, thereby ensuring data security and personal privacy. As a result, within just a few months of DeepSeek openly releasing its models, hundreds of hospitals in China announced that they had implemented local deployments. The widespread and decentralized deployment of open-source LLMs also mitigates systemic risks associated with monopolistic control, service failures, or errors from a single provider [41]. In contrast to closed-source LLMs, which rely on proprietary licensing, keep architectural and training parameters confidential, require high-performance hardware, and involve data uploads, open-source LLMs significantly promote the democratization of AI technology. This advancement facilitates the broader application of AI in health care and contributes to global AI health equity.

### Quality of Care

LLMs can evaluate the standardization level of medical images such as x-rays and computed tomography (CT) scans and generate corresponding reports. DeepSeek-R1, for instance, has demonstrated excellent performance in auditing CT reports [42]. By integrating textual descriptions with imaging data, LLMs can also generate diagnostic suggestions. Gemini 2.0 Flash has shown leading capabilities in quality control tasks for chest x-rays [43]. Through analyzing patient symptoms, electronic health records, and laboratory data, LLMs can classify chronic diseases—such as diabetes, cancer, and heart disease—and predict potential risks. For example, DeepSeek-R1 achieved 100% accuracy in mental health and oncology classification tasks [20]. Multimodal AI systems that combine CT imaging with biomarkers (eg, epidermal growth factor receptor gene mutations) have reached a 95.2% accuracy rate in distinguishing benign from malignant pulmonary nodules, surpassing the average performance of human physicians [38].

In terms of clinical reasoning and decision-making, LLMs support multistep diagnostic reasoning, such as generating examination suggestions and treatment plans. When provided with complete examination results, models such as DeepSeek-R1 achieve over 85% accuracy. However, their performance declines significantly in highly complex tasks involving examination recommendations and therapeutic planning. Evaluations on the MedR-Bench dataset reveal that LLMs still have limitations in complex scenarios and require integration with expert knowledge [23]. While LLMs demonstrate high accuracy in medical diagnosis and treatment [20,23,44], it is noteworthy that clinical decisions made solely by LLMs have, in certain cases, outperformed collaborations between physicians and LLMs [45].

Both closed-source and open-source LLMs are trained on publicly available standardized health care datasets and are dynamically updated with newly released medical guidelines and knowledge. Thus, the quality of general health services provided by both types of LLMs should be comparable. However, when open-source LLMs are deployed locally and fine-tuned using regional medical knowledge and case data, they can be adapted to highly specific medical scenarios and specialized cases. With sufficient local medical expertise, the quality of specialized health care services offered by open-source LLMs may even surpass that of closed-source alternatives.

### Cost

Closed-source LLMs require ongoing use fees (volume-based or time-based billing), whereas open-source models involve low use and licensing costs but higher initial deployment investment. Individual users can access open-source LLMs free of charge, whereas accessing closed-source models typically requires continuous payment. If APIs are called, both open-source and closed-source models may incur costs, but open-source options are generally much more affordable. Most importantly, open-source models only require a one-time deployment investment with no subsequent use fees.

Accessing the official websites of open-source LLMs is free for individual users; using the basic versions of closed-source LLMs may also be free. However, the advanced versions and complex functionalities of closed-source LLMs generally require payment. Both individuals and enterprises are charged for API calls to LLMs regardless of whether they are open-source or closed-source. However, open-source LLMs are significantly more affordable. For instance, the API cost for DeepSeek-R1 is US $0.55 per million input tokens and US $2.19 per million output tokens, which is only 6.71% of the cost of OpenAI’s GPT-o1 model [46]. Unlike the ongoing expenses associated with using closed-source LLMs, locally deploying an open-source LLM involves a one-time deployment cost rather than continuous payments to service providers. The scale of this investment depends on the size of the model being deployed and the required computational resources. Taking DeepSeek-R1 as an example, its 1.5B parameter model can even be deployed on an iPhone. In contrast, deploying the largest 671B parameter version demands substantial hardware resources—including at least 8 GPUs, a server-grade central processing unit, storage equipment, and networking infrastructure—with a minimum cost of US $1 million.

Why can open-source LLMs offer free access to their websites and low-cost API use? This is primarily because open-source LLMs benefit from significantly lower research, development, and training costs, enabling sustainable business practices. For instance, DeepSeek has substantially reduced its research and development expenses through algorithmic innovations and engineering optimizations [47]. In comparison to closed-source LLMs, the pretraining cost of DeepSeek-V3 was approximately US $5.576 million (based on H800 GPU rentals), significantly lower than the US $60 million spent on training Meta’s Llama 3.1 [48]. By adopting a mixture-of-experts architecture and efficient training strategies, DeepSeek has compressed training resource demands, reducing its training costs by over 42.5% compared to closed-source LLMs such as GPT-4 or Claude 3.5 [49]. Furthermore, open-source LLMs benefit from continuous improvements driven by the global AI community, which further enhances their efficiency and reduces expenses. Historical trends suggest that, as open-source LLM technology becomes more widely adopted and popularized, AI’s impact on health will increasingly promote equity.

## Discussion and Conclusions

Since its emergence, AI technology has been applied in the health care domain, evolving from early expert systems to the current variety of diagnostic and treatment support systems, which primarily embody the physician-AI interaction pattern. However, the widespread and direct engagement of patients with AI technology became prominent only with the advent of LLMs. This trend accelerated significantly following the open sourcing of LLMs, facilitating the patient-AI interaction pattern, where patients began using these models extensively for consultation, self-diagnosis, and even prescription information gathering. The proliferation of these interaction patterns has propelled AI health into a widespread phenomenon, marking a new and distinct phase in the evolution of digital health. In terms of accessibility, quality, and cost, the greatest advantage of open-source LLMs over closed-source alternatives lies in their ability to make both the physician-AI interaction and patient-AI interaction patterns more widespread and equitable.

Open-source LLMs not only promote AI health equity in traditional dimensions—such as across nations, regions, and populations—but also, by enabling shared access for both health care providers and patients, foster equity in a new dimension: a more balanced clinician-patient relationship within the physician-AI-patient interaction pattern. In traditional health care systems, only medical professionals hold the authority to make diagnostic and treatment decisions, whereas patients are typically excluded from participating in choices concerning their own health and disease management. A significant information asymmetry exists between physicians and patients regarding health and medical knowledge, resulting in highly unequal power dynamics during clinical decision-making.

The emergence of LLMs, especially open-source LLMs, has disrupted this imbalance. A growing number of patients now upload their laboratory results, medical images, and even full diagnostic records to LLMs seeking diagnostic interpretations and management recommendations based on their symptoms, history, and clinical data. Although the output of these models is intended for reference only, their high diagnostic accuracy—often surpassing that of average physicians—has led some patients to use LLM-generated insights to facilitate discussions with their physicians regarding diagnosis and treatment plans. Others rely on such suggestions to evaluate the appropriateness of the care recommended by their clinicians. On the health care supply side, medical providers have already begun integrating LLMs into professional workflows, making decisions based on AI-generated assessments or even fully adopting AI-proposed solutions. Introducing open-source LLM–supported diagnostics into medical decision-making enables shared decision-making between clinicians and patients based on AI-informed insights. This transforms the conventional care paradigm, leading to a more equitable relationship between patients and providers throughout the diagnostic and therapeutic process.

The technological democratization driven by open-source LLMs holds significance not only for countries such as China that are leading the open-source movement but also for global health. A core objective of global health is health equity, and addressing the growing disparities in health outcomes worldwide is a shared challenge for all nations. Open-source LLMs substantially enhance the accessibility of AI technology for both supply- and demand-side actors in health care. As long as patients have basic internet-connected devices such as smartphones, they can access open-source LLMs. Similarly, health care providers such as hospitals can deploy suitable models if they possess fundamental computing resources. This is particularly transformative for hospitals, physicians, and patients in low- and middle-income countries as open-source LLMs can effectively improve the accessibility and quality of their health services. Countries previously marginalized by expensive commercial systems can now leverage mobile apps to facilitate initial self-diagnosis [50,51], thereby advancing progress toward universal health coverage.

While open-source LLMs hold significant promise for improving AI health equity, their application in the medical field still faces considerable challenges. Open-source LLMs are prone to a certain degree of AI hallucination, making it crucial to ensure the accuracy and reliability of the medical advice they generate. Some patients may engage in self-medication based on LLM-generated recommendations [50], and errors in such advice could be life-threatening. Although open-source LLMs emphasize that their suggestions are “for reference only” and advise users to “follow professional medical guidance,” legal and ethical questions regarding accountability in cases of medical errors or malpractice remain unresolved [37]. Regarding the openness of LLMs and their diverse users, corresponding ethical and governance strategies should be established. For instance, in the case of deploying fully open-source LLMs, the responsibility for model custodianship, updates, and accountability for application outcomes lies with the deployer rather than the developer.

The comprehensive and standardized recommendations generated by open-source LLMs can mitigate information asymmetry between physicians and patients, yet they may also inadvertently undermine trust in clinical authority. Moreover, integrating even the smallest-parameter open-source LLMs into existing health information systems demands dedicated staff training and infrastructure investment. Meanwhile, the deployment of variably scaled models—such as 70B vs 671B parameter versions—contingent on local hardware capacity may perpetuate or even exacerbate disparities in AI adoption. To address these challenges, global policymakers must collaborate with stakeholders across sectors to establish robust governance frameworks for medical AI. Such frameworks should proactively tackle technical, financial, and ethical barriers, ensuring that AI-driven health services are effectively deployed worldwide and equitably accessible, thereby advancing health outcomes for all.

## Abbreviations

AI: artificial intelligence
API: application programming interface
CT: computed tomography
GPU: graphics processing unit
LLM: large language model
